# Water protection after tympanostomy (Shepard) tubes does not decrease otorrhea incidence – retrospective cohort study^☆^

**DOI:** 10.1016/j.bjorl.2017.06.009

**Published:** 2017-07-17

**Authors:** João Subtil, Ana Jardim, André Peralta Santos, João Araújo, José Saraiva, João Paço

**Affiliations:** a Universidade Nova de Lisboa, Departamento de Otorrinolaringologia, Cirurgia de Cabeça e Pescoço, Lisboa, Portugal; b Consultor de Epidemiologia e Saúde Pública, Lisboa, Portugal

**Keywords:** Otitis media with effusion, Serous otitis media, Glue ear, Tympanostomy, Otite média com efusão, Otite média serosa, Tubos de ventilação, Timpanostomia

## Abstract

**Introduction:**

Myringotomy for tube insertion is the most common otologic surgery. Otorrhea is a frequent complication of this procedure and, to prevent it, most surgeons strongly recommend avoiding contact with water as this is thought to adversely impact on post-operative quality of life.

**Objective:**

To understand the benefit of this recommendation.

**Methods:**

Observational study – retrospective cohort study comparing the incidence of post-operative otorrhea and its impact on patients’ quality of life, in two groups of patients comprising children under 10 years of age who underwent bilateral myringotomy and tube placement for chronic otitis media with effusion between May 2011 and May 2012. One group received water protection care after surgery, the other did not. Data was collected through telephonic interview, after one year of follow up (one year after the procedure). Water exposure without protection was considered the exposure event. Incidence of otorrhea and perceived impact on quality of life were the outcome measures. Results were compared after logistic regression.

**Results:**

We included 143 children: 116 were not exposed to water without protection and 27 were exposed. In the not exposed group 36.2% had at least one episode of otorrhea, compared to 40.0% of the exposed group. Odds ratio for otorrhea on exposed was 1.21 (95% CI 0.51–2.85, *p* = 0.6). Negative impact on quality of life was reported by parents of 48.2% on the not exposed children, compared to 40.7% on the exposed group. This difference was not significant (*p* = 0.5).

**Conclusion:**

We found that recommending water protection did not have beneficial effect on the incidence of otorrhea after myringotomy with tubes on chronic otitis media with effusion. However, such measures did not appear to have a negative impact on quality of life. This is a populational observational study with few cases (143 cases); these final statements would be better stated by a very large populational study with another large control group.

## Resumo

**Introdução:**

A miringotomia para inserção de tubo de ventilação é a cirurgia otológica mais comum. Otorreia é uma complicação frequente deste procedimento e, para evita-la, a maioria dos cirurgiões recomenda evitar o contato com a água, pois acredita-se que isso possa afetar negativamente a qualidade de vida pós-operatória.

**Objetivo:**

Verificar o benefício dessa recomendação.

**Método:**

Estudo observacional - estudo de coorte retrospectivo, comparando a incidência de otorreia pós-operatória e seu impacto na qualidade de vida dos pacientes, em dois grupos de pacientes com crianças menores de 10 anos submetidas à miringotomia bilateral e colocação de tubo de ventilação para o tratamento de otite média crônica com efusão, entre maio de 2011 e maio de 2012. Um grupo recebeu cuidados de proteção contra a água após a cirurgia, o outro não. Os dados foram coletados através de entrevista telefônica, após um ano de seguimento (um ano após o procedimento). A exposição à água sem proteção foi considerada o evento de exposição. A incidência de otorreia e o impacto percebido na qualidade de vida foram as medidas de resultado. Os resultados foram comparados após a regressão logística.

**Resultados:**

Incluímos 143 crianças: 116 não foram expostas à água sem proteção e 27 foram expostas. No grupo não exposto, 36,2% apresentaram pelo menos um episódio de otorreia, em comparação com 40,0% do grupo exposto. A razão de chances (*odds ratio*) para otorreia no grupo exposto foi de 1,21 (IC 95%: 0,51-2,85, *p* = 0,6). O impacto negativo na qualidade de vida foi relatado pelos pais de 48,2% nas crianças não expostas, em comparação com 40,7% no grupo exposto. Essa diferença não foi significante (*p* = 0,5).

**Conclusão:**

Não verificamos um efeito benéfico sobre a incidência de otorreia ao recomendar a proteção contra a água após colocação de tubos de ventilação para otite média com efusão. Entretanto, tais medidas não parecem ter tido um impacto negativo na qualidade de vida.

## Introduction

Otorrhea (ear drainage) is the most frequent complication of myringotomy with tympanostomy tube insertion, with an incidence of 30–83%.^1^, ^2^, ^3^ Upper respiratory tract infections are the single most common risk factor for otorrhea in children with tympanostomy tubes.^2^, ^3^

Water passing through the eardrum to the middle ear causes acute mucosal inflammation.^4^, ^5^ This has traditionally led surgeons to advise against exposure to water following insertion of tympanostomy tubes. Most surgeons prescribe ear plugs in situations where there is a risk of exposure to water, such as showering, bathing, or swimming, and some would even forbid swimming or going to the beach^1^, ^4^ to minimize the risk of post-operative middle ear inflammation.

These cases can have an impact on quality of life, not only for the children who must carry plugs and bands, or even be prevented from swimming, but also for parents and carers who must enforce these restrictions. To our knowledge this impact has not yet been assessed.

Most general practitioners and many otolaryngologists (53%) continue to recommend restricted contact with water.^1^ In 2013, we surveyed all the Hospitals in greater Lisbon, Portugal, and found that in all of them patients were advised to avoid contact with water following tympanostomy tube placement (this survey was published in the Portuguese Otolaryngology Society Meeting in 2014).

However, there is growing evidence in recently published epidemiological studies, that water does not cross tympanostomy tubes unless under significant pressure (corresponding to diving deeper than 60 cm in water).^2^, ^5^, ^6^ However, most of these studies present multiple limitations and confounding factors, with only one randomized controlled trial (RCT) presenting grade B evidence,^3^ and the remaining few being observational studies. This RCT concludes there is evidence of some statistically significant benefits with the routine use of ear plugs, which contradicts previous observational studies. The most recent guidelines from the American Academy of Otolaryngology on tympanostomy tubes does not recommend routine use of ear plugs^7^ and a recent review by Cochrane concludes the same, albeit stating that the overall quality of the body of evidence is low.^8^

The inconsistencies found in the literature and the absence of studies assessing the impact on quality of life of such precautions, led us to conduct the present study.

The primary question we used was whether the incidence of otorrhea episodes after myringotomy and transtympanic tubes was higher in children without ear plugs when exposed to water (e.g. swimming, showering), than in children with ear plugs during such activities. The secondary question was whether avoiding contact with water had a negative impact on quality of life on both populations.

## Methods

### Study design and setting

We conducted a retrospective cohort study developed at a Portuguese private hospital in Lisbon, Portugal. It included a population of children between 2 and 10 years old, admitted for bilateral myringotomy with tube placement and adenoidectomy, between May 1st, 2011, and May 1st, 2012.

### Study population

All children were operated on the same hospital, and the eligibility criteria included: (1) age < 10; (2) indication for surgery following diagnosis of chronic Otitis media with effusion (OME) (ICD-10 code H65.2, H65.3, H65.4) in accordance with American Academy of Otolaryngology guidelines for OME; (3) Bilateral tube placement and adenoidectomy; Sheppard tube; and (4) follow-up longer than one year.

Patients with a diagnosis other than OME (e.g. recurrent otitis media), who had previous surgery (myringotomy and/or tube placement surgery and/or adenoidectomy), or received a tube other than Sheppard (e.g. Goode) were excluded. Other exclusion criteria included a follow-up period less than a year after surgery (or lost for follow-up), lack of records on personal clinical file or drop out.

### Study intervention and outcome

The participants included in this study where operated by 9 (nine) different surgeons, who prescribed homogeneous post-operative care, except for ear protection when subjected to water exposure. All of them were allowed water exposure, but 7 (seven) surgeons always prescribed ear canal protection when water exposure was expected (not exposed to the intervention), while the remaining two did not (exposed to the intervention). The prescriptions reflect surgeon's personal preferences at the time of surgery, and this study did not interfere with their choice. This difference allowed the constitution of two cohorts, the exposure being defined as not prescribing ear canal protection from water exposure after surgery.

The level of prescribed care was checked on the medical record and confirmed by the parents. As we also wanted to understand how compliant the participants were regarding the level of care prescribed, they were asked how thorough they were about protecting the ears (plugs, headband), and how deep they were diving (e.g. just surface swimming or diving deep too).

The primary outcome variable was otorrhea episodes. It was used as a surrogate for middle ear inflammation. The question was “how many episodes of ear drainage during the first year” (0, 1, 2–4, >5) (primary outcome variable). Patients did not have a specific log book for these events, but were all instructed to write in their health log book and to inform the surgeon whenever any relevant event happened, which in turn was logged on the patients’ medical record.

The secondary outcome variable was impact on quality of life; we studied the covariable “level of impact on quality of life caused by precautions taken” and “level of impact on quality of life by prescribed precautions (if different)”.

The study was authorized by the Hospital Ethics Committee (doc. CEHCD.ORL1-26092012), and the parents gave consent following a telephonic interview. A trained investigator conducted the interviews and questionnaires. Data was obtained from medical records and telephonic questionnaires, with multiple choice questions. Parents completing the questionnaire were asked to check the child's health log book for clinical events (in Portugal every child has a health log book, kept by the parents, where both parents and doctors record relevant clinical events).

Each question addressed one covariable of the study. The covariables were: “age at time of surgery”, “gender” and “upper airway tract infection episode”.

The covariable “upper airway tract infection episode” addressed the coexistence of upper airway infection symptoms with acute otorrhea episode. These included blocked nose, rhinorrhoea, sore throat, or any other symptom in this area suggestive of at least one of the following: rhinitis, sinusitis, adenoiditis, tonsillitis, pharyngitis, laryngitis, epiglottitis or tracheitis. With this question, we want to query a possible association of acute otorrhea episode with upper airway infection.

To assess otorrhea episodes following exposure to water, we studied the covariable “episodes related to water passing into the ear”.

To characterize the otorrhea episodes as to their duration and severity, we asked respectively “how long did it last most of the times” (<1 d, 1 d, 2–3 d, 4–7 d, 7 d); and “treatment” (e.g. oral antibiotics, drops, paracetamol/acetaminophen, other anti-inflammatory treatment, other treatment).

### Statistical analysis

Statistical treatment was carried out with STATA 13 software. Descriptive analysis included absolute and relative frequencies, Chi-square (*x*^2^) to compare proportions (categorical variables), and *t*-Student to compare averages. Inferential analysis used logistic regression, with non-adjusted and adjusted odds ratio. The level of significance was *p* < 0.05.

## Results

### Population characteristics

**Figure 1:**
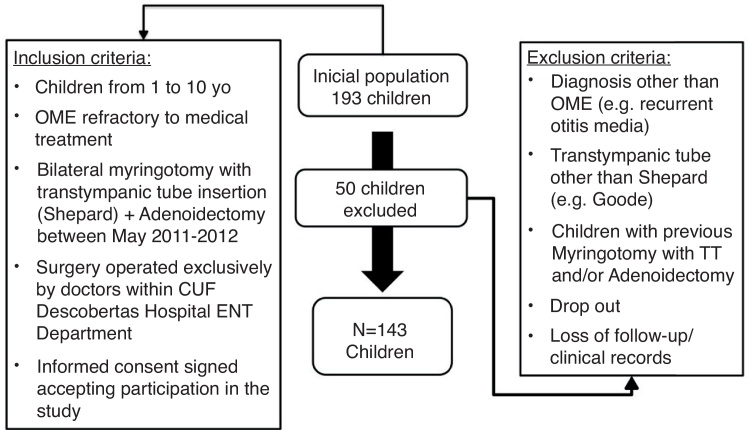
Inclusion and exclusion criteria.

After enrolling 193 children, only 143 met the inclusion criteria and were not excluded (Fig. 1). All 143 children included had a follow-up appointment at least 12 months after the surgery. For 116 patients, surgeons prescribed ear plugs. We included these 116 patients in Group B designated ‘Not exposed’. The remaining 27 patients were advised not to wear ear plugs and were included in Group A, designated ‘Exposed’ to the intervention.

The average age at the time of surgery was 3.8 years in the group without protection (A) and 4.2 in the group with protection (B). This was considered not to have significant statistical difference (*p* = 0.07) between the groups. The gender in the exposed and non-exposed group also did not differ significantly (*p* = 0.5). The two cohorts were considered homogeneous regarding “age at time of surgery”, “gender” and “upper airway tract infection episode”, as shown in Table 1.

**Table 1:** Baseline population characteristics.

	Without protection (*n* = 27)	With protection (*n* = 116)	*p*-Value
Age (mean and SD)	3.8 (1.68)	4.2 (1.23)	0.07
Gender (male) (%)	63	56	0.6
UAI (%)	22.2	2.7	0.8
Antibiotics (%)	33.3	34.5	0.9
Otorrhea (%)	40.0	36.2	0.6
Negative impact on quality of life (%)	40.7	48.2	0.5

*Note*: Otorrhea episodes were related to upper respiratory tract infection on 4 (36.4%) subjects in Group A, and 21 (48.8%) in Group B (46.3% on both groups together). On Group A, 16 felt no impact and 11 felt negative impact (1 very little impact, 2 little impact, 1 felt so-so, 2 felt much impact, and 5 very much). On the not exposed Group B, 60 felt no impact and 56 felt negative impact (7 very little impact, 8 little impact, 18 felt so-so, 9 felt much impact, and 14 very much).

In Group A (exposed/without protection), 16 patients had no episodes of otorrhea; the rest of the group had at least one episode in the study period. In Group B (not exposed/with protection), 74 patients did not have any episodes of otorrhea, with the remaining patients having had at least one episode. Almost all episodes lasted less than one day. Treatment comprised oral and topical antibiotics in 8 (72.7%) affected subjects on Group A, and in 35 (83.3%) patients in Group B.

Interestingly, when we split patients in Group A into two additional categories, comparing the ones who of were just swimming but not diving deep, with the ones who swam and dived deep, we found that only 2 out of a total of 11 not diving participants (18.2%) of the first group had at least one episode, versus 9 out of 16 (56.3%) of the diving ones. This has no statistical significance because of the small number of participants, but suggests a difference when not diving deep.

### Otorrhea incidence

The odds ratio of having at least one episode of otorrhea in Group A patients (‘Exposed’/Not using ear protection) was 1.21, with a 95% confidence interval of 0.51–2.85 (Table 2). This indicates that not wearing protection poses a slightly higher risk, but the difference between both groups was deemed not statistically significant, with *p* = 0.6.

**Table 2:** Non-adjusted odds ratio of having otorrhea episode or quality of life impact for the group with protection prescribed.

	OR	95% CI	*p*-Value
*Otorrhea (1st outcome)*			
Without protection	1.21	0.51–2.85	0.6

*QOL (2nd outcome)*			
Without protection	0.74	0.31–1.72	0.5

Otorrhea episodes were related to upper respiratory tract infection on 4 (36.4%) subjects in Group A, and 21 (48.8%) in Group B (totalling 46.3% in both groups).

When adjusted (Table 3), the odds of having otorrhea in Group A were 1.59 (with 95% CI 0.50–5.04) and remained non-significant. Interestingly, age and male gender had an odds ratio bellow 1, and so, although not significant, it suggests that being older and male is a protective factor.

**Table 3:** Adjusted odds ratio of having otorrhea episode or quality of life impact for the group with protection prescribed.

	OR	95% CI	*p*-Value
*Otorrhea (1st outcome)*			
Without protection	1.59	0.50–5.04	0.4
Age at time of surgery	0.75	0.51–1.10	0.1
Gender (male)	0.56	0.21–1.59	0.3

*QOL (2nd outcome)*			
Without protection	0.85	0.34–2.14	0.7
Age at time of surgery	1.31	1.01–1.72	0.05
Gender (male)	1.62	0.79–3.33	0.2

On the other hand, having an upper airway infection (UAI) was associated with an odds ratio of having otorrhea of 136.1 (with a 95% CI 16.23–1141.6 and *p* < 0.001). This odds ratio is not surprising considering that otorrhea is surrogate of UAI.

### Quality of life

As to the secondary outcome, the level of impact on quality of life, on the exposed Group A, 16 patients felt no impact and 11 felt a negative impact (1 negligible impact, 2 slight impact, 1 felt moderate, 2 felt significant, and 5 severe). In Group B (not exposed/with protection) 60 patients felt no impact and 56 felt a negative impact on their quality of life (7 negligible impact, 8 slight impact, 18 felt moderate, 9 felt significant, and 14 severe). The odds ratio of feeling any negative impact on quality of life when exposed to not having been prescribed water protection was 0.74 (with a 95% CI 0.31–1.72) (Table 2), indicating that not wearing protection would cause less impact, though not reaching significance, as the difference between both groups showed *p* = 0.5 significance, even after adjustment for age and gender (Table 3).

## Discussion

In our retrospective study, we compared two cohorts of participants, one wearing protection whenever exposed to water (Group B) and the other (Group A) without this protection.

We found that having been prescribed water protection or avoidance after myringotomy surgery with tubes apparently did not have a relevant impact on quality of life, and that not taking such precaution did not lead to increased frequency of otorrhea.

As we have seen before, most primary care physicians and many otolaryngologists (53%) continue to recommend avoidance of contact with water^1^ even after one randomized study by Goldstein et al. in 2005, which suggested this was not necessary. According to Wilcox in 2014, there is still a lack of consensus^9^ in this matter, mainly due to a lack of strong evidence of the benefit of either recommending or not such protections.

There is growing evidence in the literature available, both in vitro as well as in epidemiological studies, that water is not crossing tympanostomy tubes unless under significant pressure (corresponding to a depth of more than 60 cm in water) because of its length and narrow calibre.^2^, ^5^, ^6^

Also, otorrhea after tube insertion has been mostly associated with upper respiratory tract infections,^2^, ^3^ but many episodes will have no apparent relation to a relevant event, and this might be because live bacteria are present in most effusions even before surgery.^10^

By enrolling all the children operated during one year we overcame the bias of having seasonal exposure to risk factors for otorrhea, namely during the winter a higher exposure to upper airway infections, and during the Summer, a higher exposure to water while swimming.

Also, because we enrolled only patients diagnosed with otitis media with effusion, we avoided the cases that would have an expected higher incidence of otorrhea, such as surgery for recurrent otitis media, which is more prone to produce episodes of otorrhea which are unrelated to water exposure, potentially masking the episodes caused by water.

This study has some limitations: we used telephonic interviews, relying on parent's recollection of events that took place at least one year before, potentially leading to recall bias. However, in Portugal, every child has a log book to record clinical events where a parent may easily find such information, and this was used to minimize this bias.

Our sample size is also limitation of our study. A greater sample size would allow us to reduce the variability and uncertainty of the results; hence some of the results should be interpreted with caution. Another limitation was grading the parent's response on non-validated scales, and this was in part because we had no specific scale for assessing impact on quality of life of wearing ear protection devices. On the other hand, we wanted to keep the questionnaire as simple as possible to facilitate the parents’ cooperation with the telephonic interview.

We found no statistical significant difference on the incidence of otorrhea between the groups. This is not surprising, since we know from previous studies that it is mechanically difficult for water to cross a tube.^2^, ^5^, ^6^

This is, as far as we know, the first study addressing the issue of impact on quality of life when prescribing water protection after myringotomy surgery for OME treatment. We used a simple question and a grading scale of six degrees, which is clearly insufficient to analyze such a complex issue, but still the answer may give us a clue and anticipate a further study. We were expecting a negative impact when imposing wearing ear plugs and head band whenever going for a swim, but instead we found no significant difference. This may be explained by the relative ease with which most children adapt to new routines, and both children and parents accept it well thinking of it as a trade-off between getting better and still be allowed to swim.

Oral antibiotics are not currently recommended as first line treatment^7^ for otorrhea, but the fact that many of the episodes were associated with an upper respiratory infection may account for the high number of cases treated with oral antibiotics.

One might argue that without relevant benefit from the protection, it would be better to stop recommending it and so we support the recent position of the American Academy of Otolaryngology.^7^

## Conclusion

We did not find that recommending water protection had a beneficial effect on the incidence of otorrhea after myringotomy with tubes for otitis media with effusion. Both groups with and without protection had a high incidence of 40.0% and 36.2%.

Also, in this study such measures did not appear to cause impact on quality of life. These results support the recent AAO guideline on tympanostomy tubes, that recommends not to encourage routine use of water protections after tympanic tube implantation. We think that more evidence, as supported by this study, will further encourage adherence to AAO guideline.

This is a populational observational study with few cases (143 cases); these final statements would be better stated by a very large populational study with another large control group.

## Conflicts of interest

The authors declare no conflicts of interest.
